# Effect of Hand and Rotary Instruments on the Fracture Resistance of Teeth: An In Vitro Study

**DOI:** 10.3390/dj8020038

**Published:** 2020-04-29

**Authors:** Nisha Acharya, Md Riasat Hasan, Dashrath Kafle, Anil Chakradhar, Takashi Saito

**Affiliations:** 1 Department of Conservative Dentistry and Endodontics, Kathmandu University School of Medical Sciences, Dhulikhel Hospital, Dhulikhel 45200, Nepal; menishaacharya@gmail.com (N.A.); anil254413@gmail.com (A.C.); 2 Division of Clinical Cariology and Endodontology, Department of Oral Rehabilitation, School of Dentistry, Health Sciences University of Hokkaido, Hokkaido 061-0293, Japan; t-saito@hoku-iryo-u.ac.jp; 3 Department of Orthodontics, Kathmandu University School of Medical Sciences, Dhulikhel Hospital, Dhulikhel 45200, Nepal; dashrath07@yahoo.com

**Keywords:** endodontic treatment, fracture resistance, pericervical dentin, rotary instruments

## Abstract

**Objective:** Endodontic treatment should be both conservative and effective. Endodontic instruments with a greater taper are used for coronal flaring, for proper debridement with efficient irrigation. However, increased taper of an instrument can remove a larger amount of pericervical dentin, compromising the strength of the tooth. The aim of this study was to determine the effect of hand files, ProTaper Universal, ProTaper Next, and V Taper rotary instrument systems on the fracture resistance of teeth. **Materials and Methods:** In total, 60 extracted human maxillary first premolars were divided into four groups—Group I (Hand Files; HF), Group II (ProTaper Universal; PT), group III (ProTaper Next; PTN) and Group IV (V Taper; VT) (N = 15). Each group was instrumented with the respective instrument system, irrigated, obturated, restored, and mounted in cold cure acrylic. A universal load-testing machine (Shimadzu, Japan) was used to apply a vertical compressive load. The maximum force was recorded in Newton. Analysis of variance (ANOVA) and Independent *t*-tests were applied to compare the maximum mean force required to fracture the tooth. **Results:** There was a statistically significant difference in fracture resistance between Group I (HF) and Group II (PT) and between Group II (PT) and Group IV (VT) (*p* < 0.001). Similarly, a significant difference was observed between Group II (PT) and Group III (PTN) (*p* < 0.01). Furthermore, a significant difference was observed between Group I (HF) and Group III (PTN), and between Group III (PTN) and Group IV (VT) (*p* < 0.05), too. However, there was no statistically significant difference between Group I (HF) and group IV (VT) (*p* > 0.05). **Conclusion:** Rotary files with more taper seem to remove more pericervical dentin than traditional manual and rotary files with less taper, thus altering the strength of the tooth.

## 1. Introduction 

Endodontically treated teeth are considered to have a lower survival rate compared to vital teeth. One of the various causes of failure of root-filled teeth is root/tooth fracture. According to Vire, nearly half of the failures of root-treated teeth are due to crown fracture (59.4%), which he describes as a prosthetic failure [1]. It has also been noted that endodontically treated teeth are extracted more frequently for non-endodontic rather than endodontic causes [2,3,4]. Moreover, the strength, integrity and manner of force distribution in the remaining tooth structure during mastication also has an influence on the long-term survival of the tooth [5].

The survival rate is found to be high in young patients due to a larger amount of remaining tooth structure after crown placement in endodontically treated teeth [6]. Although the reasons are multifactorial, the loss of bulk of the tooth structure during root canal treatment plays a major role in the long-term survival of the tooth. Thus, preservation of a larger amount of tooth structure not only increases fracture resistance, but also maintains the structural integrity of post-endodontically-restored teeth [7,8,9,10]. The various rotary endodontic instruments with new blade designs and increased taper need an extended access cavity and early coronal flaring to maintain straight-line access into the middle third of the root canal [11]. The critical pericervical dentin (PCD), i.e., the dentin roughly 4 mm above and 4 mm below the alveolar crest, is the point of force-concentration responsible for tooth fracture. This irreplaceable dentin near the crestal bone reinforces the tooth and is crucial for strengthening it [12]. However, variably tapered instruments remove larger amounts of this critical cervical dentin, resulting in significant weakening of the tooth root [13,14,15].

Most new rotary endodontic instruments incorporate instruments with a taper greater than the international organization for standardization (ISO) standard of 2%. The ProTaper Universal system (Dentsply Maillefer, Ballaigues, Switzerland) consists of partially active tips in shaping and non-cutting tips in finishing files with a progressive taper to reduce the torsional loading [16]. Shaping files S1 and S2 have 0.17 and 0.20 mm tip diameters with taper ranging from 0.02 to 0.11 and 0.04 to 0.115 between D1 and D14, respectively. Finishing files F1, F2 and F3 have respective D0 diameters of 0.20, 0.25 and 0.30 mm, and a taper of 0.70, 0.80 and 0.90 between D0 and D3, respectively. However, these files have a decreasing percentage of tapers from D4 to D14 [17].

The ProTaper Next (PTN) rotary files (Dentsply, Maillefer, and Ballaigues, Switzerland) utilize M wire technology with progressive tapers on each file and an off-centered rectangular design [18]. There are 5 PTN files available, X1, X2, X3, X4 and X5, corresponding to tip diameters of 17, 25, 30, 40 and 50 mm, respectively. X1 files have a taper of 0.04 at D1, 0.05 at D3, 0.065 at D6, 0.075 at D9, and 0.06 between D13 and D16. Similarly, X2 files have a taper of 0.06 between D1 and D3, 0.07 between D6 and D9, 0.06 at D13, and 0.04 at D16. However, the PTN X3, X4 and X5 files consist of a fixed taper of 0.075, 0.065 and 0.06, respectively, from D1 to D3, then a decreasing percentage tapered design over the rest of the active portions of their cutting length, reaching 0.050, 0.045 and 0.04 at D16, respectively [17]. 

The V taper system (SS White, Philadelphia, USA), made of a modified NiTi alloy called Endonol, has a multi-taper design and a reduced-diameter shaft for flexibility. It consists of a series of three variable tapered files, with tip diameters of 0.20, 0.25 and 0.30 mm respectively, and a 0.06, 0.08 and 0.10 taper at D0–D4, respectively. Moreover, V taper (VT) (20) has a 0.03 taper, VT (25) a 0.04 taper, and VT (30) a 0.05 taper from D4 to D8, respectively, and a 0.02 taper from D8 to D12 in all three files [19]. 

Hence, this study was conducted in order to discover the effect of manual files, and ProTaper Universal, V Taper and ProTaper Next rotary files, on the fracture resistance of teeth.

## 2. Materials and Methods

This study was conducted in accordance with the declaration of Helsinki, and the protocol for this research was obtained from Institutional Review Committee—Kathmandu University School of Medical Sciences (IRC-KUSMS), Dhulikhel Hospital, on 7 March 2019, with approval number 70/19. Sixty intact, single-rooted maxillary first premolars, with 18 ± 2 mm root length and two root canals, were collected from orthodontic patients undergoing therapeutic extraction, with an age group of 18 to 24 years. Immediately after extraction, teeth were immersed in 3% sodium hypochlorite solution for 5 min and ultrasonic scalar was used to remove soft tissues, calculus, and any external debris from the teeth, before examination by two experienced endodontists for detection of any cracks, fractures or hypocalcification/hypoplasia under magnification (loupe, 3.5×) and illumination. Radiographs of all the specimens were taken from proximal (mesial and distal) and buccal aspects for the detection and exclusion of teeth with caries, fracture/craze lines, developmental anomalies, immature/open apices, thin curved root/severe apical curvature, multiple canals, calcification, more than one root/two canals, and previous restoration or endodontic treatment. The specimens meeting the inclusion criteria were blindly allocated into 4 experimental groups—Group I (Hand File Group; HF), Stainless Steel Hand K files (Dentsply, Maillefer Switzerland); Group II (ProTaper Universal Group; PT), ProTaper Universal Rotary NiTi files (Dentsply, Maillefer, Switzerland); Group III (ProTaper Next Group; PTN), ProTaper Next Rotary NiTi files (Dentsply, Maillefer, Switzerland); and Group IV (V Taper Group; VT), V taper Rotary NiTi files (Guidance Endodontics, Albuquerque, NM, USA). Samples were then stored in four different containers using 10% buffered formalin solution until further use. 

Each tooth was accessed with Endo access burs (SS White, Philadelphia, USA), and size #10 and #15 hand K files were used to establish a canal patency and working length determination, respectively, in the entire specimen. The working length was set at 0.5 mm short of the apical foramen. With an estimated working length and in the presence of Ethylenediaminetetraacetic acid (EDTA), (Glyde, Dentsply, Maillefer Switzerland) as a chelator and lubricant, all 4 experimental groups were instrumented in accordance with the manufacturer’s protocol at recommended speed and torque settings. In Group I, canals were instrumented with hand K files up to ISO size #30 (0.30 mm tip diameter and 0.02 taper), and preparation completed with ISO size #50 (0.50 mm tip diameter and 0.02 taper) using the step-back technique. Group II specimens were prepared with ProTaper rotary files in a sequence of S1, S2, F1, F2 and F3, in a crown-down manner. SX files were not used. Similarly, Group III specimens were instrumented with ProTaper Next rotary files in a sequence of X1, X2 and X3, using the full-length technique. Subsequently, Group IV specimens were instrumented with V Taper rotary files in a sequence of V20, V25 and V30 until the working length was achieved. Recapitulation was done with #15 hand K files and 17% EDTA (MD-Cleanser, Metabiomed), and 3% sodium hypochlorite (NaOCl, Pyrex, India) was used; 3 ml in each canal with each sequential instrument was used as an irrigant during the preparations of all the specimens.

**Figure 1:**
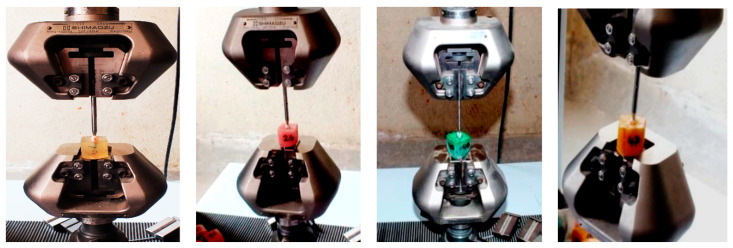
Load testing of the sample (Groups I, II, III and IV, respectively).

**Figure 2:**
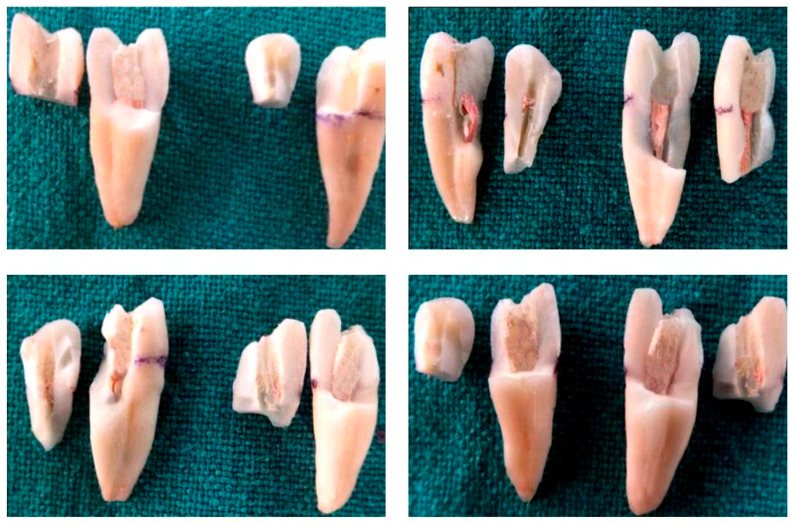
Samples after load testing in Groups I, II, III and IV, respectively.

Canals were dried with sterile paper points and coated with resin-based sealer (Biofill Root canal sealer, Medicept dental, UK). The gutta percha points of the respective instrument systems of corresponding size and taper were used for obturation. The lateral condensation technique was used to obturate the samples from Group I, the master cone being #30. Several lateral cones were also used in this technique to obturate the canals properly. However, the single cone obturation technique was utilized for Group II (master cone F3), Group III (master cone X3) and Group IV (master cone V30), without any accessory cones. The samples were then stored in a humidifier maintaining 100% humidity at room temperature to facilitate the setting of the sealer. After 48 h, the specimens were restored with a Filtek Z 250 Universal restorative resin composite (3M, ESPE, USA). The root surface of each entire sample was coated with a thin layer of modeling wax (Pyrex, India), 2 mm below the cervical line, to produce a 0.2 to 0.3 mm layer, using a carver in order to simulate the periodontal ligament. A square wax block of 3 x 3 cm was used as a template, and auto-polymerizing acrylic resin (Pyrex, Rapid Repair, Roorkee, India) was mixed with water color to produce 4 different colors. The teeth were then vertically aligned, with the long axis of the tooth being parallel to that of the wax block, and mounted 2.0 mm apical to the cement–enamel junction in an acrylic resin. The wax spacer was removed from the root surface and alveolus of the acrylic resin block using hot water. Vinyl polysiloxane light body impression material (Aquasil, Dentsply, Germany) was mixed and applied into the acrylic resin in the alveolus space created via the de-waxing procedure. The samples were then reinserted into the resin blocks, and the impression material was allowed to set. The fracture resistance of the samples was then tested using a Universal Testing Machine (Shimadzu, Japan) with Trapeziumx operating software. Compressive load under a constant crosshead speed of 1 mm/min was applied until a sudden drop in the value appeared (Figure 1 and Figure 2). The force required to fracture each tooth was recorded in Newton (N).

Statistical analysis was performed using Statistical Package of the Social Sciences (SPSS) version 17.00 (SPSS Inc., Chicago IL). One-way analysis of variance (ANOVA) and Independent/unpaired *t*-tests were applied to compare the mean fracture loads of the groups and determine the significance of differences between different groups. The level of significance for all the statistical tests was determined at 0.05.

## 3. Results 

**Figure 3:**
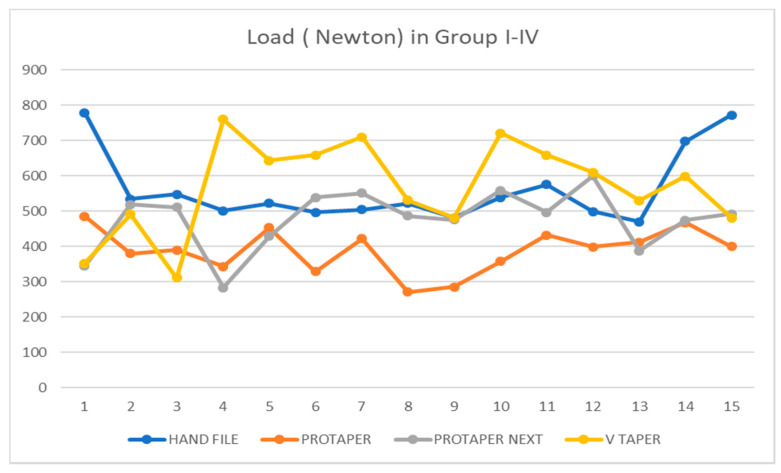
Graphical representation of pattern of load distribution in different groups.

The pattern of load distribution in all four experimental groups is shown in Figure 3. The mean load required to fracture the tooth, standard deviation and comparison are illustrated in Table 1. The results showed a statistically significant difference among different groups in fracture resistance (*p* ≤ 0.001). Furthermore, the intergroup comparison showed a statistically significant difference between HF and PT (*p* ≤ 0.001), HF and PTN (*p* = 0.017), PT and PTN (*p* = 0.003), PT and VT (*p* ≤ 0.001), and PTN and VT (*p* = 0.029). However, there was no statistically significant difference between HF and VT (*p* = 0.880).

## 4. Discussion

The key factor determining the fate of a root-treated tooth is fracture predilection, which directly depends on the amount of tooth tissue loss [20]. Approximately 7.5% of endodontically treated teeth are extracted within five years post-treatment, which mostly includes molars and premolars [21]. The reasons for extraction include vertical root fracture (13.4%) and non-restorable crown and cusp fracture (15.1%), in addition to periodontal problems (40.3%) and endodontic failure (19.3%) [22]. Vire found three major categories of failure (prosthetic, periodontal and endodontic origin) among root-treated teeth [1]. Out of these, 59.4% had prosthetic failures primarily due to crown fracture, 32% due to periodontal failures, and 8.6% due to endodontic causes.

Moreover, Zandbiglari et al. suggested that excessive coronal enlargement with increased tapered instruments significantly weakens the tooth, leading to fracture [15]. Hence, our study attempted to evaluate the relation between the variable taper of three rotary endodontic instruments, i.e., ProTaper, ProTaper Next and V Taper, as well as hand files, and their influence on the fracture resistance of a tooth as a whole. The amount of PCD removed in order to accommodate the traditional access cavity and the variable taper of the rotary instruments might have a cumulative effect on the fracture resistance of teeth. One of the most important findings of the present study is that the VT group exhibited the highest mean fracture load (569.35 ± 131.26 Newton). This observation may be attributed to the decreasing percentage of taper of VT files, resulting in a narrow diameter at the cervical area compared to that of other rotary file system used in this study. All three VT rotary files used in this study have a 0.02 taper from D8 to D12 [19], resulting in the preservation of the PCD. The preservation of the crucial PCD to the greatest possible extent has been suggested to reinforce and increase the fracture resistance of a tooth [12,23]. Moreover, the mean force required to fracture a tooth was lowest for PT (388.57 ± 62.46 Newton). Although the PT finishing files have less taper coronally, shaping files S1 and S2 incorporate tapers of 0.11 and 0.115, respectively, at D14 [16]. Kunert et al. reported that the taper is one of the main factors in apical root transportation and unnecessary removal of dentin [24]. The statistically significant difference observed between our experimental groups could be the consequence of an increased taper of PT and PTN files, compared to traditional ISO standardized 2% tapered hand K files and less tapered VT files. Similarly, Bier et al. found more dentinal defects when canals were instrumented with PT (16%), compared to Profile (8%) and Greater Tapered (4%) files [25]. This could explain the low fracture resistance of PT files compared to other file systems. In contrast to previous findings, Lam et al. found that the fracture resistance of roots is not reduced by a greater enlargement, or by an increased taper, of an instrument any degree more than the conventional K file [26]. In our study, a significant difference was found between HF and PTN files as well, which can again be attributed to the larger amounts of dentinal crack formation and dentine removal, leading to the reduced fracture resistance of a tooth by greater-tapered instruments [24,25]. In a study conducted by Prati et al., approximately 80% of root-treated teeth survived and were functional for more than 20 years [2]. This high survival rate may be attributed to the use of traditional hand files, along with the use of bonded restoration and the skill of the operator. In the present study, Group I (Hand Files) required the highest mean load to fracture, which is a finding similar to those of previous studies.

However, we found no statistically significant difference between hand and VT, which is a similar result to that of the study done by Akhlaghi et al. [19]. Additionally, in this study, most of the fractures occurred at 3–4 mm below the cervical line, which indicates the importance of preserving PCD, as mentioned clearly by Clark and Khademi [23,27]. Similarly, Ricks-Williamson et al. found that the highest stress magnitudes were located between the middle and coronal thirds of the root, an area prone to fracture during treatment [28]. Hence, one of the most important aspects of increasing the survival rate of endodontically treated teeth is the preservation of the remaining sound tooth structure to the greatest possible extent.

## 5. Conclusions

Within the limitations of this in vitro study, it is concluded that hand files and rotary files with less taper (V Taper) seem to better preserve the strength of teeth than rotary files with increased tapers (ProTaper Universal and ProTaper Next). Hence, clinicians should be careful when choosing instruments for root canal treatment and should be as conservative as possible, provided that the objective of root canal treatment is met.

## Figures and Tables

**Table 1:** Comparison of mean load (Newton) among different groups.

Groups	Mean	Standard Deviation	ANOVA (F)	Sig. (*p*)	Intergroup Difference (*p* value)					
I (HF)	562.82	101.79	11.242	*p* ≤ 0.001 ***	HF/PT	HF/PTN	HF/VT	PT/PTN	PT/VT	PTN/VT
II(PT)	388.57	62.46								
					*p* ≤ 0.001 ***	0.017 *	0.880	0.003 **	*p* ≤ 0.001 ***	0.029 *
III(PTN)	476.49	84.54								
IV(VT)	569.35	131.26								

HF: Hand Files; PT: ProTaper; PTN: ProTaper Next; VT: V Taper. * Significant, ** Very Significant, *** Highly Significant.

## Data Availability

The data used to support the findings of this study are included within the article.
